# Cell-Free and Yeast-Based Production of the Malarial Lactate Transporter, PfFNT, Delivers Comparable Yield and Protein Quality

**DOI:** 10.3389/fphar.2019.00375

**Published:** 2019-04-10

**Authors:** Philipp Hajek, Annika Bader, Folknand Helmstetter, Björn Henke, Philipp Arnold, Eric Beitz

**Affiliations:** ^1^Department of Pharmaceutical and Medicinal Chemistry, Pharmaceutical Institute, Christian-Albrechts-University Kiel, Kiel, Germany; ^2^Anatomical Institute, Christian-Albrechts-University Kiel, Kiel, Germany

**Keywords:** cell-free protein production, *Pichia pastoris*, membrane protein, transport, *Plasmodium falciparum*, L-lactate, inhibitor binding, biolayer interferometry

## Abstract

Cell-free protein production is an attractive alternative to cell-based expression. Rapid results, small-volume reactions, irrelevance of protein toxicity, flexibility, and openness of the system are strong points in favor of the cell-free system. However, the *in vitro* situation lacks the cellular quality control machinery comprising e.g., the translocon for inserting membrane proteins into lipid bilayers, and chaperon-assisted protein degradation pathways. Here, we compare yield and protein quality of the lactate transporter, PfFNT, from malaria parasites when produced in *Pichia pastoris* yeast, or in an *Escherichia coli* S30-extract-based cell-free system. Besides solubilization and correct folding, PfFNT requires oligomerization into homopentamers. We assessed PfFNT folding/oligomerization and function by transmission electron microscopy imaging, transport assays, and binding of small-molecule inhibitors. For the latter, we used chromatography of the PfFNT-inhibitor complex with dual-wavelength detection, and biolayer interferometry. Our data show, that PfFNT possesses an intrinsic capability for assuming the correct fold, oligomerization pattern, and functionality during *in vitro* translation. This competence depended on the detergent present in the cell-free reaction. The choice of detergent further affected purification and inhibitor binding. In conclusion, in the presence of a suitable detergent, cell-free systems are very well capable of producing high quality membrane proteins.

## Introduction

The production and purification of membrane proteins for functional and structural studies remains a tedious endeavor. It requires a wise choice of a suitable expression system in order to obtain sufficient quantities and adequate quality for the purpose. Further, practicality and economic parameters typically need to be taken into account, such as costs for culture media and required volumes. In this regard, cells that can be grown in suspension exceed by far the mass-per-volume yield of adherent cells, in particular when a fermenter is used. Consequently, the amount of extracted membrane protein can be expected to be considerably higher. Common suspension cultures for the production of membrane proteins are based on bacteria, mainly *Escherichia coli*, yeast, such as *Saccharomyces cerevisiae* (Hannig and Makrides, 1998; Jidenko et al., 2005; Pedersen et al., 2007) or *Pichia pastoris* (Cereghino and Cregg, 2000; Cregg et al., 2000; Long et al., 2005; Törnroth-Horsefield et al., 2006), or insect cells, e.g., *Spodoptera frugiperda* Sf9 (Jasti et al., 2007). Probably the largest protein yield in relation to reaction volume can be obtained if cells are completely omitted by using an *in vitro* transcription/translation system. The basis of such a cell-free protein production setup system is a cell-extract that contains mainly the translation machinery, in particular the ribosomes from a suitable prokaryotic or eukaryotic donor species. All additionally required components, such as RNA and protein building blocks (nucleotides, amino acids, and tRNAs), enzymes, and energy regeneration systems need to be supplied and should not be limiting during the reaction (Schwarz et al., 2007). Another yield-affecting factor is the accumulation of metabolic end products that should be continuously removed from the reaction in order not to slow down enzymatic conversion rates or to negatively act on the concentration-dependent steady state equilibria (Schwarz et al., 2007).

In this study, we produced the malarial lactate transporter, PfFNT (Marchetti et al., 2015; Wu et al., 2015), i.e., a homopentamer of 150 kDa total mass, in two systems with putatively high protein yield, i.e., fermenter cultures of *P. pastoris* yeast, and a continuous-exchange cell-free system (CECF) based on an S30 *E. coli* extract.

*Pichia pastoris* offers the possibility to produce particularly high amounts of protein for mainly two reasons. First and foremost, the cells can be grown to very high densities yielding 400 g of wet cells per liter (Cereghino et al., 2002). Second, methanol induction of protein expression is done when the desired cell density is reached ensuring high-level expression independent of a possibly negative growth effect of the target protein (Tschopp et al., 1987). This way, several milligrams of purified membrane protein can be anticipated per liter of *P. pastoris* culture. The yield is influenced by the nature of the produced protein, however, in particular membrane proteins can have toxic effects on the cell physiology even without being explicit toxins. Further, the cells are capable of adding post-translational modifications on the expressed protein, and typically preserve intact protein when frozen and stored at -80°C, which constitute additional advantages (Tschopp et al., 1987).

A continuous exchange cell-free system can produce several milligrams of protein per milliliter in an overnight reaction (Kigawa et al., 1999; Holm-Bertelsen et al., 2016). Therefore, small cell-free sample volumes of 1–3 ml can yield comparable amounts of protein as 2–5 L of *P. pastoris* fermenter cultures. The cell-free system is open and amenable to direct manipulation of the transcription and translation steps. For instance, prosthetic groups and isotope-labeled amino acids for NMR structure analyses can be added (Kigawa et al., 1999), or unnatural amino acids can be incorporated, e.g., by the amber stop codon-suppression technique (Wals and Ovaa, 2014). In the case of membrane proteins, provision of a lipid compartment (liposomes and nanodiscs) or detergent is indispensable if precipitation of the protein should be prevented (Berrier et al., 2004; Klammt et al., 2005; Katzen et al., 2008). The direct solubilization of the nascent membrane protein chain by detergent typically produces higher yields than a liposome or nanodisc setup. However, the yield comes at the cost of an artificial environment, i.e., a detergent micelle, that may mimic the natural lipid membrane environment less well. Compared to cell-based systems, direct translation of the protein into detergent micelles offers the opportunity for instantaneous purification without additional operation steps, such as solubilization and centrifugation. A convenient way to identify suitable detergents for the cell-free reaction is by analytical-scale screenings using a C-terminal fusion construct of the membrane protein with green fluorescent protein (GFP) at the C-terminus as a fluorescent *in situ* folding indicator (Müller-Lucks et al., 2012).

**FIGURE 1:**
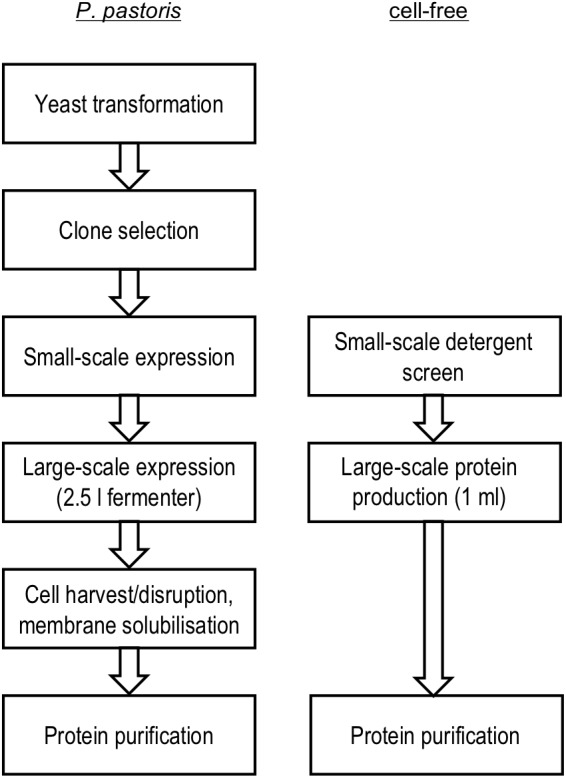
Workflow PfFNT production in *P. pastoris* yeast cells and in an *E. coli* S30 extract cell-free setup with of detergent present.

Cell-based and cell-free systems, thus, differ greatly in terms of handling, costs, time, and in assisting correct protein folding. For comparison, we employed *P. pastoris* fermenter cultures and an *E. coli* S30 extract-based cell-free system in detergent mode (see Figure 1 for the used workflow) and assessed the yield and quality of PfFNT aiming at later structure determination. We characterized the produced PfFNT using diverse techniques, such as direct imaging by transmission electron microscopy, measuring transport functionality by stopped-flow light-scattering, and determining inhibitor binding by size-exclusion co-purification and biolayer interferometry.

## Materials and Methods

### Cell-Free Production of PfFNT

We used an *E. coli* S30 extract-based cell-free system to produce PfFNT as described earlier (Holm-Bertelsen et al., 2016). T7 polymerase-driven transcription was done from a pIVEX2.3 plasmid harboring a codon-optimized open reading frame for PfFNT (Supplementary Figure S1A). The expression construct further encoded an N-terminal hemagglutinin-tag, and a C-terminal factor Xa digestion site followed by a His_10_-tag. PfFNT protein production was done in reaction volume of 1 ml using a dialysis cassette (Slide-A-Lyzer, Thermo Fisher Scientific) with a 10 kDa cut-off (Thermo Fisher Scientific). The reaction mix contained all necessary ingredients, such as the plasmid DNA, T7-polymerase, nucleotides, the *E. coli* S30 extract, t-RNAs, amino acids, and an energy regeneration system (Schwarz et al., 2007). The reaction was supplemented with 0.8% polyoxyethylen (Müller-Lucks et al., 2012) stearyl ether (Brij 78) for direct solubilization of the nascent PfFNT membrane protein chain (detergent-containing cell-free mode, DCF). The dialysis cassette was placed into 17 ml of feeding-mix, which served as donor compartment for nucleotides and amino acids, and as a sink for waste metabolites. The reaction was kept at 30°C for 20 h in a shaking water bath.

### Yeast-Based Production of PfFNT

For production of PfFNT in *P. pastoris* yeast, we used the PichiaPink expression system (Thermo Fisher Scientific; Supplementary Figure S1B). The cells were batch-cultured in 100 ml of buffered glycerol-complex medium (for composition see Bushell et al., 2003) to accumulate biomass to an OD_600_ of 25–35 at 30°C. Fermentation was done by adding 2 L of buffered glycerol-complex medium in a 5 L-fermenter at 30°C. A minimal oxygen concentration of 30% was kept by regulating the stirring speed, and pH 5 was maintained by supply of acid (HCl) or base (NaOH) for 24 h. Expression of PfFNT was induced indirectly via upregulation of alcohol oxidase by steadily adding 400 ml of methanol over 40 h. The cells were harvested by centrifugation at 4000 *g* for 10 min at 4°C, yielding about 500 g of cell mass. Cells were disrupted in portions of 80 g (wet weight) using equal volumes of acid washed glass beads and cell disruption buffer (25 mM Tris–Hcl, pH 7.5, 5 mM EDTA) in a beadbeater (BioSpec) with 10 intervals of 30 s each on ice. The suspension of disrupted cells was cleared at 5,000 *g* and 4°C for 10 min, and the membrane fraction was collected at 200,000 *g* and 4°C for 45 min. The membrane proteins were solubilized with 4.5% N,N-dimethyldodecyl-amine N-oxide (LDAO) in 20 mM TRIS, pH 7.5, 200 mM NaCl, 10% (v/v) glycerol overnight at 4°C with a magnetic stirrer. The buffer volume was adapted to yield 5 mg ml^-1^ of total protein.

### Purification of PfFNT

Solubilized PfFNT (35 ml from yeast expression, or 2 ml cell-free reaction) was incubated overnight with 333 μl Ni-NTA bead slurry (Qiagen) using a rotating mixer at 4°C. The slurry loaded on columns and was washed with 20 volumes of 20 mM imidazole-containing buffer of 20 mM TRIS, pH 8.0, plus 200 mM NaCl, 10% (v/v) glycerol and 0.05% Brij 78, 0.17% n-decyl-β-D-maltopyranoside (DM), 0.05% n-dodecyl-β-D-maltopyranoside (DDM), 1.2% n-octyl-β-D-glucoside (β-OG), or 0.27% LDAO as indicated. PfFNT was eluted in fractions of 5 column volumes with increasing imidazole concentrations of 80–500 mM. Fractions with the highest PfFNT content were pooled and subjected to size exclusion chromatography using a Superdex 200 10/300 GL column (GE Healthcare) in the purification buffer without imidazole with a constant flowrate of 0.35 ml min^-1^, 1.2 MPa at 4°C.

### Negative Staining Electron Microscopy and Class Sum Formation

Negative staining was performed as described before (Arnold et al., 2014), however, half saturated uranyl acetate was used. After transfer of samples into a JEOL1400 Plus transmission electron microscope operating at 100 kV, images were taken on a TVIPS F416 digital camera binned to 2048 × 2048 pixel. The nominal magnification was 50.000, resulting in a resolution of 4.58 Å/pix. Single particles were selected, aligned and summed using EMAN2 (Tang et al., 2007). Here the graphical user interface (e2projectmanager.py) was used to organize the individual steps for image analysis. For the alignment process the reference free alignment was used without any symmetry imposed.

### Reconstitution of PfFNT Into Proteoliposomes and Transport Analysis by Stopped-Flow Light Scattering

Reconstitution of PfFNT was done as described previously (Holm-Bertelsen et al., 2016). Briefly, 25 mg of *E. coli* polar lipids (Avanti) were dissolved in chloroform, evaporated under a nitrogen stream, and dried under vacuum. The lipid film was hydrated for 1–2 h at room temperature in liposome buffer (5 mM HEPES/MES, pH 6.8, 100 mM KCl, 2 mM 2-mercaptoethanol, 200 mM sucrose) to yield a final lipid concentration of 50 mg ml^-1^. The samples were shock-frozen in liquid nitrogen, thawed, and sonicated to obtain multilamellar liposomes. 100 μg of purified PfFNT were added at a protein-lipid ratio of 1:50. Reconstitution was forced by 25-fold dilution with liposome buffer. The proteoliposomes were harvested by ultracentrifugation at 140,000 *g* and 4°C for 45 min, resuspended in 1 ml liposome buffer, and passed 21 times through a 0.2 μm filter (LiposoFast extruder, Avestin). L-Lactate transport into proteoliposomes was monitored at 20°C by changes in 524 nm light scattering using a stopped flow device (SFM-2000, Bio-Logic Science Instruments) by rapid exposure to a 200 mM inward substrate gradient. In this setup, the liposomes initially shrink by osmotic efflux of water increasing the light scattering signal intensity, and subsequent re-swelling initiated by the slower influx of L-lactate decreases the light scattering intensity. The obtained signal traces were averaged (*n* = 3–9) and normalized. Transport rates were calculated from double-exponential fitting (Bio-Logic software).

### Synthesis of Fluorescent and Biotinylated PfFNT Inhibitors BH697 and BH565

BH697 and BH565 were generated in four chemical synthesis steps. First, ethyl pentafluoropropanoate and 4-hydroxyphenylacetophenone were treated with LiH in anhydrous tetrahydrofuran to yield [4,4,5,5,5-pentafluoro-3-hydroxy-1-(4-hydroxyphenyl)pent-2-en-1-one] (Sosnovskikh and Usachev, 2001). This compound was alkylated in the 4-position using *tert*-butyl *N*-(3-bromopropyl) carbamate in the presence of NaH in anhydrous dimethylformamide (Inman et al., 2014). The *tert*-butyloxycarbonyl protecting group was cleaved with trifluoroacetic acid in dichloromethane (Bourguet et al., 2016). The ammonium moiety of the resulting intermediate product was treated in diisopropylethylamine and dimethylformamide with either 5(6)-carboxyfluorescein-*N*-hydroxysuccinimide ester to yield BH697, or with biotin-*N*-hydroxysuccinimide to obtain BH565 (Nakamura et al., 2014).

### Co-purification of PfFNT With Fluorescent Inhibitor

Purified PfFNT was incubated for 30 min at 20°C with 10 μM of the fluorescence-labeled MMV007839 inhibitor compound BH697. The PfFNT-inhibitor-complex was separated from unbounded inhibitor by size exclusion chromatography (Superdex^®^ 200 10/300 GL) with a dual-wavelength photometric detector (280/490 nm). Protein fractions with bound inhibitor were pooled and used for repeated separations over 24 h. The relative amount of bound BH697 was determined by relating the absorption by the inhibitor measured at 490 nm to the absorption of the protein at 280 nm.

### Biolayer Interferometry

The interaction between the small-molecule inhibitor MMV007839 (Golldack et al., 2017) and yeast-based or cell-free produced PfFNT was done by biolayer interferometry (Octet RED96e, FortéBio). MMV007839 carrying a biotin moiety, BH565, was bound to streptavidin-coated sensors (SA sensor, FortéBio) in 20 mM Tris, pH 8.0, 150 mM NaCl, 1% DMSO for 25 min. Unreacted streptavidin-sites were blocked by adding 10 μg ml^-1^ biocytin for 10 min. For monitoring association, 300 nM solutions of PfFNT in purification buffer were added to the inhibitor-coated sensors and biolayer interference followed for 20 min at room temperature and shaking at 1000 rpm. For dissociation, the sensors were placed in buffer of the same composition yet without protein with a shaking speed of 500 rpm. Samples of yeast-produced PfFNT additionally contained 0.5% bovine serum albumin. Streptavidin-coated sensors without the MMV007839 inhibitor, yet blocked with biocytin served as controls and obtained biolayer interference signals were subtracted from the samples. The derived traces were fitted using the 2:1 model of the Octet System Data Analysis Software.

## Results

### Production and Purification of PfFNT

Cultivation of 2.5 L of *P. pastoris* in a fermenter yielded 460 ± 31 g wet cells. In the following, we used cell portions of 80 g for cell disruption, membrane protein solubilization and purification steps. The remaining cell mass was storable at -80°C for later use. In the search for a mild, yet efficient detergent for membrane protein solubilization we ended up using LDAO because it provided higher yields and more stable PfFNT homopentamers than alternative detergents, such as DM, DDM, fos-choline 12, CHAPS, or β-OG. The choice of detergents was made with respect to their suitability in later crystal screens.

Cell-free production of PfFNT was done overnight in 1 ml batches using dialysis cassettes in a feeding solution for continuous exchange of nutrients and waste. Nascent PfFNT protein was directly solubilized by Brij 78 detergent that was present in the reaction. We chose Brij 78 because we identified this detergent before as being highly suitable for obtaining high yields of correctly folded PfFNT in the cell-free process (Holm-Bertelsen et al., 2016). Since PfFNT was contained as solubilized protein in the reaction mix, it was directly usable for protein purification.

**FIGURE 2:**
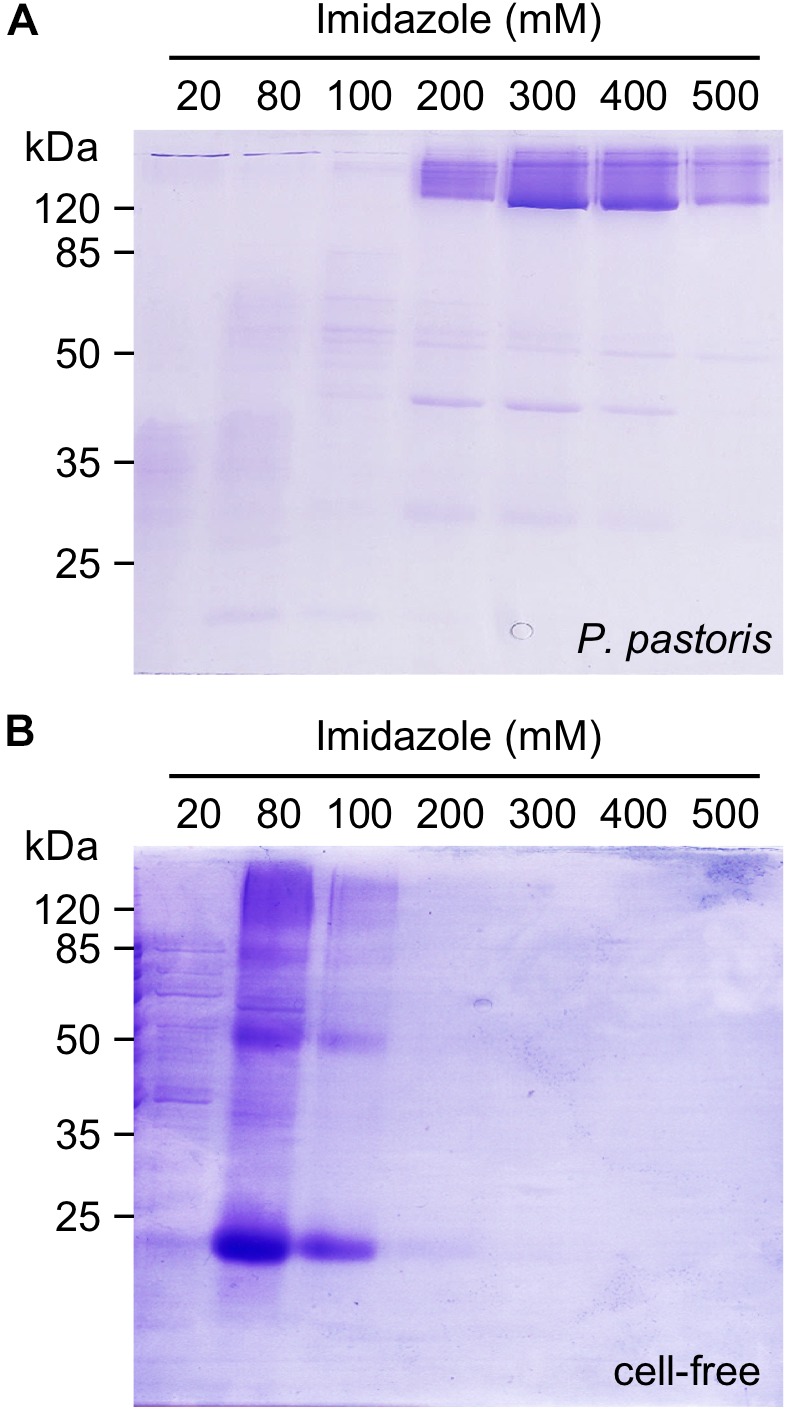
Ni^2+^-affinity purification of *P. pastoris* expressed PfFNT in DM **(A)** and cell-free produced PfFNT in Brij 78 **(B)** via C-terminal His-tags. The protein was solubilized with DM in the case of cell-based expression or with Brij 78 in the cell-free system. PfFNT monomers have a molecular mass of 30 kDa, the pentamer accordingly 150 kDa.

Making use of a C-terminal His_10_-tag, we bound the cell-based as well as the cell-free produced PfFNT protein to Ni^2+^-nitrilotriacetate beads (NTA) for immobilized metal affinity purification (Figure 2). During purification, the solubilizing detergent was exchangeable. We successfully eluted the yeast-produced and Ni^2+^-bound PfFNT with LDAO, β-OG, and DDM (Supplementary Figure S2). We obtained the highest PfFNT yields in DM containing elution buffers, which occurred above 200 mM imidazole with most intense PfFNT bands in the 300 and 400 mM imidazole fractions (Figure 2A). Despite the presence of SDS in the gels, PfFNT largely retained an oligomeric structure as seen by the bands above the 120 kDa marker. We isolated PfFNT pentamers from the combined 300 and 400 mM imidazole fractions by size-exclusion chromatography yielding 1.4 ± 0.3 mg in total. Cell-free, Brij 78-solubilized PfFNT was eluted at lower, 80–200 mM imidazole concentrations and appeared as a protein ladder containing monomers up to pentamers (Figure 2B). The observed downshift in affinity might be explained by the larger size of the Brij 78 polar head groups that may partially conceal the His-tags. We attempted to exchange Brij 78 for DM and DDM during affinity purification yielding virtually the same outcome with respect to SDS-resistance (Supplementary Figure S3). This is indicative of a low tendency of Brij 78 micelles to exchange with other detergents. The combined elution fractions from the cell-free reactions typically contained around 1 mg of PfFNT protein, i.e., an amount comparable to extracting 80 g of *P. pastoris* yeast.

### Cell-Based and Cell-Free Produced PfFNT Form Stable, Solubilized Pentamers

**FIGURE 3:**
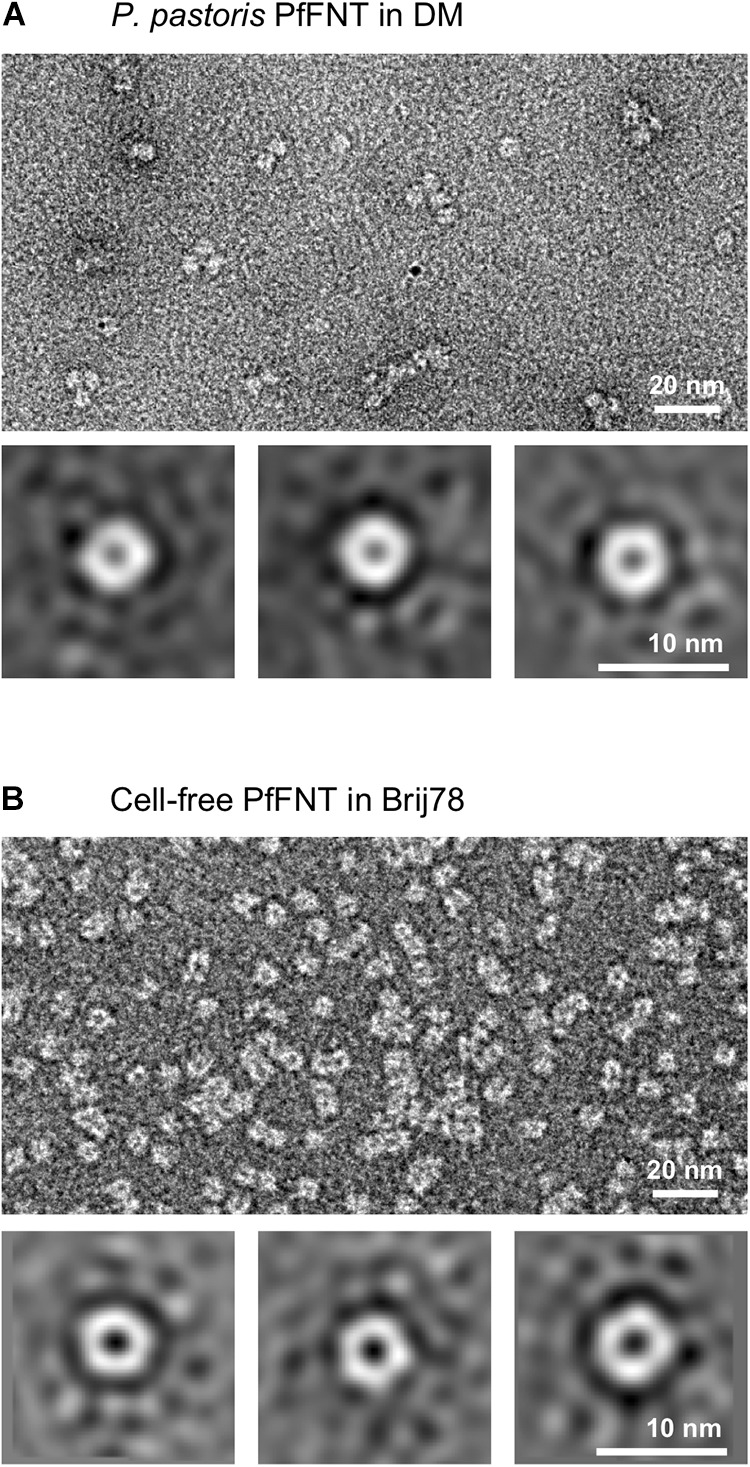
Direct imaging of *P. pastoris* expressed PfFNT in DM **(A)** and cell-free produced PfFNT in Brij 78 **(B)**. The DM detergent facilitated some agglomeration of the particles, whereas Brij 78 led to more even dispersion of individual particles. Class summations depict pentamer formation in both samples.

To test whether the apparently different SDS-resistance of yeast-derived and cell-free produced PfFNT affects homopentamer stability under less harsh conditions, we applied transmission electron microscopy (TEM) to directly image samples of cell-based and cell-free PfFNT (Figure 3).

Both samples displayed an overall even distribution of particle sizes. In the *P. pastoris* cell-based PfFNT sample, the particles tended to agglomerate (Figure 3A). This behavior was dependent on the used detergent, because the degree of agglomeration decreased upon replacement of DM (however at the cost of signal-to-noise ratio, see Supplementary Figure S4). Accordingly, the Brij 78-solubilized, cell-free PfFNT depicted well dispersed particles (Figure 3B). Particle class summations revealed a consistently pentameric PfFNT structure for both samples (Figure 3, lower panels). This observation is particularly striking for the cell-free produced PfFNT, which never encountered a native lipid bilayer environment, and, thus, indicates an intrinsic structural self-organization capability of the FNT proteins.

In the following, we set out to further evaluate the quality of the PfFNT protein samples derived from cell-based *P. pastoris* and cell-free production by assaying transport functionality and inhibitor binding.

### Cell-Based and Cell-Free Produced PfFNT Proteins Are Functional Lactate Transporters

**FIGURE 4:**
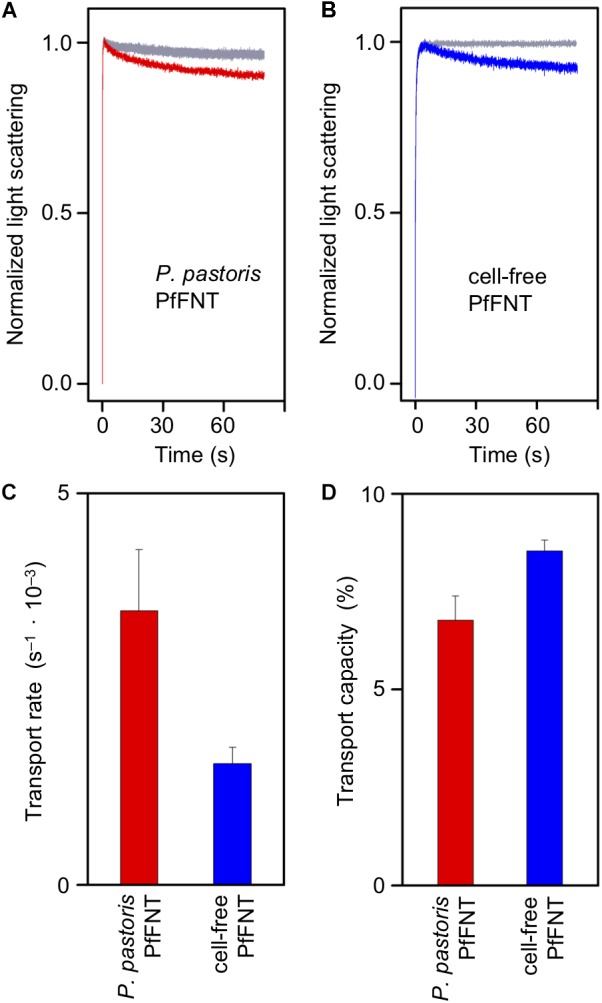
Transport of L-lactate of *P. pastoris* expressed PfFNT in DM (red) and cell-free produced PfFNT in Brij 78 (blue) by stopped-flow light scattering. The proteins were reconstituted into proteoliposomes and challenged by a 200 mM osmotic L-lactate gradient. **(A,B)** The bi-phasic curves show rapid liposome-shrinkage due to osmotic water efflux, and subsequent, slower lactate uptake via PfFNT. Empty control liposomes (gray) do not transport lactate. **(C)** Rates of PfFNT lactate transport were calculated from bi-exponential fitting of the light scattering traces. **(D)** The capacity of transport is given as volume re-gain after osmotic shrinking and lactate uptake.

We reconstituted purified *P. pastoris* cell-based and cell-free produced PfFNT into proteoliposomes following a protocol that we established earlier (Müller-Lucks et al., 2012; von Bülow et al., 2012; Golldack et al., 2017; Erler et al., 2018; Helmstetter et al., 2019). We evaluated PfFNT transport functionality by challenging the proteoliposomes with an inward directed, osmotic lactate gradient of 200 mM. Under these conditions, the proteoliposomes will shrink in an initial rapid phase due to the osmotic release of water across the lipid bilayer. If the integrated PfFNT protein is functional and facilitates lactate import the proteoliposomes will partially regain volume on a slower time scale. We followed the changes in proteoliposome volume by monitoring 90° light scattering with a stopped-flow apparatus. Liposome shrinking results in an increase in light scattering, whereas swelling decreases the light scattering intensity. Accordingly, plain liposomes without PfFNT protein maximally shrank within the first second and then maintained an even signal level (Figure 4, gray traces). PfFNT-containing proteoliposomes also showed the initial osmotic shrinking effect but subsequently the light scattering signal decreased due to re-swelling by lactate import (Figure 4A,B). The cell-based and the cell-free PfFNT preparations exhibited similar transport properties indicating functional protein in either case.

Exponential fitting of the bi-phasic light scattering traces yielded about two times higher transport rates of lactate import with the yeast-produced, DM-solubilized PfFNT protein than with cell-free PfFNT in Brij 78 (Figure 4C). The capacity of transport was similar, though (Figure 4D). This is indicative of a higher degree of integration of the yeast-derived PfFNT and may be due to the used detergent. However, at this point it cannot be excluded that the cell-free preparation contains a larger proportion of non-functional, misfolded protein. Therefore, we decided to looked at complex formation between PfFNT and a small-molecule inhibitor serving as an indicator for correct folding of the binding site, which is located deep in the PfFNT transport path (Golldack et al., 2017).

### Cell-Based and Cell-Free Produced PfFNT Equally Bind a Small-Molecule Inhibitor

**FIGURE 5:**
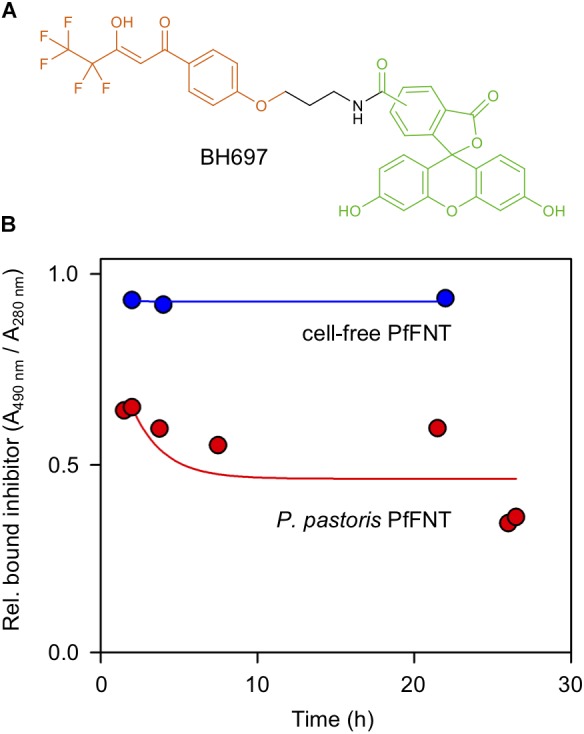
Binding of a fluorescence-labeled PfFNT inhibitor during size exclusion chromatography. **(A)** Chemical structure of the inhibitor BH697. The molecule is composed of the actual screening-derived PfFNT inhibiting moiety (orange), an amide linker (black), and carboxyfluorescein (green). **(B)** Stability of cell-based (red) and cell-free PfFNT-BH697 complexes (blue) over time. The proportion of bound inhibitor (l = 490 nm) was related to the PfFNT protein content (l = 280 nm).

The small-molecule FNT inhibitor MMV007839 (Golldack et al., 2017; Erler et al., 2018) exhibits an unusually high, quasi-covalent binding affinity to PfFNT, i.e., even after prolonged washing for several hours the compound remained bound to the PfFNT protein (Golldack et al., 2017). Therefore, we investigated long-term binding of the inhibitor to the purified PfFNT samples to test for an intact and correctly folded interaction site in the protein. We incubated PfFNT for 30 min with a 10 μM concentration of the inhibitor MMV007839 to which we had covalently bound a carboxyfluorescein moiety via an amide linker (BH697; Figure 5A). We decided on the linker attachment site by investigations on the structure-activity relationships which showed that MMV007839 was highly tolerant to modifications at the methoxy group (Golldack et al., 2017). The samples containing PfFNT and BH697 were then repeatedly passed over a size exclusion column to remove unbound inhibitor and to relate the bound BH697 to the PfFNT protein by integrated dual-wavelength absorption (l_BH697_ = 490 nm / l_protein_ = 280 nm).

Again, cell-based and cell-free produced PfFNT behaved quite similar. In either case, after the first passage over the size exclusion column the inhibitor remained stably bound to PfFNT over the full observation interval of 24 h (Figure 5B). The initial drop after the starting time point (time 0 h), which appeared larger in the *P. pastoris*-produced, DM-solubilized PfFNT (45 vs. 5%), most likely can be attributed to loosely bound unspecific inhibitor.

### Cell-Based and Cell-Free Produced PfFNT Exhibit Similar Inhibitor Binding Kinetics

To kinetically resolve the phase of complex formation between PfFNT and the small-molecule, we employed biolayer interferometry (BLI) which allows one to observe interactions in real-time. In a typical BLI application, the protein of interest is coupled to a reflecting light sensor and subsequently dipped into the inhibitor solution to monitor binding events via interference of the reflected white light. We started out by biotinylating cell-based and cell-free produced PfFNT and coupling of the protein to streptavidin-coated sensors. Despite good loading efficiency (interference phase shift of about 6 nm), subsequent exposure to the MMV007839 inhibitor solution did not produce signals above the noise level. Hence, this approach was not feasible, and it is generally difficult when using membrane proteins because the size of the solubilizing detergent micelles strongly limits the density of immobilized protein at the sensor surface.

**FIGURE 6:**
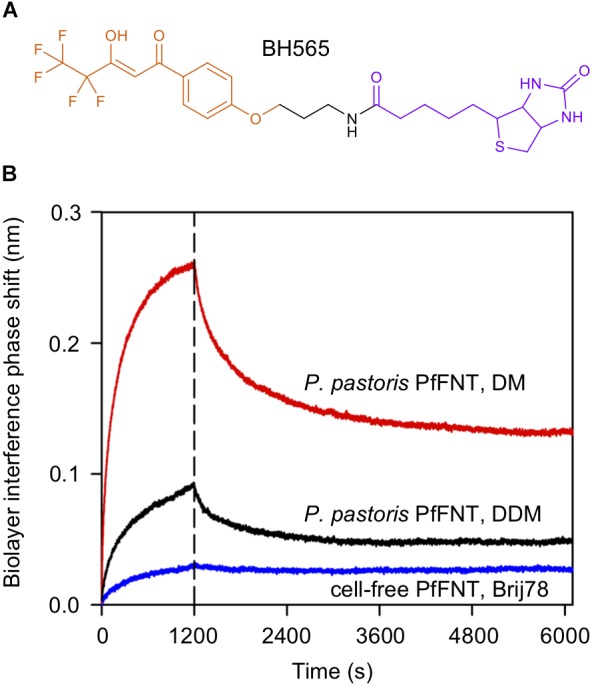
Assessment PfFNT binding to an immobilized PfFNT inhibitor by biolayer interference. **(A)** Chemical structure of the inhibitor BH565. The molecule is composed of the actual screening-derived PfFNT inhibiting moiety (orange), an amide linker (black), and biotin (violet). **(B)** BH565 was bound to streptavidin-coated light sensors and binding (0–1200 s) as well as dissociation (>1200 s) of solubilized cell-based, DM-solubilized PfFNT (red), cell-based, DDM-solubilized PfFNT (black), and cell-free, Brij 78-solubilized PfFNT (blue). The detergent affected the binding capacity (amplitude at 1200 s) and the degree of unspecific bound inhibitor (rapid drop after 1200 s).

We therefore reversed the setup of the assay by binding the small-molecule inhibitor to the sensor. To this end, we synthesized a biotinylated variant of MMV007839, which we termed BH565 (Figure 6A), for coating streptavidin BLI sensors. The inhibitor-loaded sensors specifically bound PfFNT derived from *P. pastoris* expression (Figure 6B, red trace, 0–1200 s) as well as from cell-free production (blue trace); weak background signals were determined with protein-free sensors and subtracted. Despite the difference in the binding amplitude, the on-kinetics were similar at 9000 ± 600 M^-1^ (cell-based, DM-solubilized PfFNT) and 6000 ± 2400 M^-1^ (cell-free, Brij 78-solubilized PfFNT). These numbers, however, were calculated based on the molecular mass of the PfFNT pentamer without taking into account the additional mass produced by the detergent micelle. If one considers that Brij-micelles can be expected to be larger than DM micelles (Schefer et al., 1988; Slotboom et al., 2008), and thus exhibit slower diffusion rates the binding kinetics become even closer. After the 1200 s binding period, we placed the sensors in PfFNT-free buffer to monitor dissociation of the inhibitor from the protein. In the dissociation phase, we observed the same behavior as in the size exclusion chromatography approach (see section “Cell-Based and Cell-Free Produced PfFNT Equally Bind a Small-Molecule Inhibitor”). About 40% of the inhibitor rapidly dissociated from the cell-based PfFNT and then maintained a stable level of PfFNT-inhibitor-complex, whereas with cell-free PfFNT the amount of non-specifically bound inhibitor was <5%.

We figured that both, the obtained signal amplitude in the binding phase (Figure 6, 0–1200 s) as well as the degree of non-specific binding of the inhibitor (Figure 6, signal drop after 1200 s) may depend on the used detergent in particular on the size of the micelle rather than different degrees of correct folding of the protein samples. Hence, we exchanged the DM detergent of the cell-based PfFNT to DDM, which forms micelles that are typically larger by about one third (Schefer et al., 1988), and re-evaluated inhibitor binding by BLI interference. Indeed, with this change we found intermediate values for the signal amplitude and for non-specific inhibitor binding (Figure 6, black trace). Detergents of the Brij-type carry particularly large polar head groups which greatly increase the micelle diameter and the load of bound bulk water even more (Slotboom et al., 2008). This experiment shows that larger detergent micelles tend to (i) lower the binding capacity of the sensor surface, and (ii) decrease the non-specific association of small drug-like molecules.

Together, our data, i.e., direct TEM structure imaging, transport functionality, and inhibitor binding, show that cell-free produced PfFNT exhibits comparable quality to PfFNT expressed in a *P. pastoris* cell-based system. Production in detergent cell-free mode, thus, constitutes a suitable alternative for structural and functional studies of membrane proteins.

## Discussion

The central aim of the study was to evaluate the quality of a membrane protein in terms of structural and functional properties when produced in a lipid- and cell-free system. In the tested case of PfFNT, also the formation of a quaternary structure was necessary, i.e., an oligomer with the correct number of five protomers. In contrast, a cell-based expression system harbors quality control measures at various levels. Already in the translation process at the rough endoplasmic reticulum the complex cellular machinery of the translocon ensures that the nascent membrane protein chain is directly and properly integrated into the lipid bilayer. Misfolding or incorrect lipid integration will expose hydrophobic domains of the membrane protein which are detected by chaperones. Subsequently, such proteins will be subjected to degradation. The cell-free system is devoid of such processes. Here, protein folding is solely facilitated by detergent maintaining the protein chain in solution, and the folding process completely depends of the structural self-organization capability of the membrane protein.

Today, multiple examples exist showing that complex membrane proteins assume their correct fold during cell-free production in the presence of detergent, chaperones, liposomes, or nanodiscs (Junge et al., 2010; Rath et al., 2011; Chi et al., 2016; Focke et al., 2016; Shinoda et al., 2016; Vaish et al., 2018; Jacobs et al., 2019). Many studies carried out cell-free membrane protein production even in precipitation mode, i.e., in the absence of a suitable detergent or lipid environment, and nevertheless successfully folded the precipitated protein in a subsequent renaturation step (Junge et al., 2010; Rath et al., 2011; Focke et al., 2016; Shinoda et al., 2016). Using these approaches, various classes of membrane proteins such as receptors [G-protein coupled (Junge et al., 2010; Chi et al., 2016; Shinoda et al., 2016; Jacobs et al., 2019); tyrosin kinase (Shinoda et al., 2016)], channels and transporters [aquaporins Shinoda et al., 2016; ion channels Shinoda et al., 2016; Vaish et al., 2018, ionotropic receptors Shinoda et al., 2016; mechanosensitive channels (Jacobs et al., 2019); bacterial porins (Junge et al., 2010); formate-nitrite transporters (Holm-Bertelsen et al., 2016; Helmstetter et al., 2019)], or cell adhesion proteins (claudins Shinoda et al., 2016) were successfully produced. Furthermore, quality assessment by functionality assays, ligand binding, and structure determination was positive in many cases. In these instances, the sequence-contained information sufficed to correctly fold the respective membrane protein. Still, adequate reaction conditions need to be provided in the process.

We found that a highly valuable tool in the analytical detergent-screening phase of the cell-free process is the production of membrane proteins carrying a green-fluorescent protein at the C-terminus as a folding indicator (Müller-Lucks et al., 2012). In order to fluoresce, the GFP must mature and form a fluorophore from three consecutive amino acid residues in a spontaneous secondary chemical reaction (Iizuka et al., 2011). The time scale of the reaction is in the several minutes to hour range depending on the used GFP. The reaction can only take place if the three residues are correctly arranged which requires native and soluble GFP. Therefore, if a membrane protein/GFP fusion exhibits fluorescence this indicates that the protein was well stabilized by the detergent providing prolonged time to assume a proper fold. The fluorescence signal can easily be visualized via in-gel fluorescence imaging of an SDS–PAGE.

In our hands, detergents of the Brij-family carrying polyoxyethylen polar head groups turned out to be most successful in facilitating cell-free membrane protein production. For stabilizing tetrameric aquaporins and pentameric FNTs, in particular Brij 58 and Brij 78, both carrying 20 ethylene glycol units, and C16 or C18 alkane chains, respectively, were most suitable (Müller-Lucks et al., 2012, 2013; Holm-Bertelsen et al., 2016; Helmstetter et al., 2019). We further successfully employed DDM with a di-saccharide polar head group (von Bülow et al., 2012). Our confirmation by TEM that homo-oligomer formation takes place under these conditions renders the cell-free technique even more powerful. With respect to later crystallization trials, the sturdiness of Brij micelles may be a disadvantage because exchange with detergents that are better suitable for crystal formation appears inefficient. It is remarkable, however, that Brij 78 gave a particularly good background and signal-to-noise ratio in protein electron microscopy. Yet, Brij-type detergents are hardly known and used in this field and for this reason an auspicious detergent class may have been missed.

The pentameric PfFNT protein carries five individual lactate transporting units (Wiechert and Beitz, 2017; Wiechert et al., 2017), i.e., one in each protomer. The central pore is impermeable to water and solutes because it is filled with lipids when the protein is present in the native environment (Wang et al., 2009). We found that cell-based and cell-free PfFNT when incorporated into proteoliposomes exhibited typical and similar transport properties in the stopped-flow assay suggesting that the central pore was closed in either case. The cell-free PfFNT, however, faced lipids only during reconstitution. Yet, it is thinkable that lipids entered the central pore in the process. Alternatively, detergent molecules may have occupied the central pore inhibiting water and substrate passage. Besides their solubilizing function and folding-support, the detergents may, thus, further serve roles in the proper functionality of cell-free produced membrane proteins.

The detergent further directly affected specific and unspecific binding of a small-molecule PfFNT inhibitor as well as the loading capacity of the light sensor in the BLI assay. Here, the chemical makeup and the size of the resulting detergent micelle most likely are key factors.

Together, rather than focusing on protein yield alone when optimizing a cell-free process for membrane protein production, the choice of detergent must not be underestimated. A suitable detergent can provide membrane protein samples of similar quality as derived from cell-based expression for meaningful structural and functional studies.

## Author Contributions

PH expressed PfFNT in *P. pastoris*, purified the protein, reconstituted proteoliposomes, conducted the cell-based PfFNT transport assays, and carried out size exclusion chromatography of PfFNT-inhibitor complexes. AB produced cell-free PfFNT, purified the protein, and measured inhibitor binding kinetics using biolayer interferometry. FH prepared *E. coli* S30 extract, produced cell-free PfFNT, purified the protein, reconstituted proteoliposomes, assayed transport of the cell-free PfFNT. BH synthesized and analyzed the small-molecule inhibitor compounds. PA prepared the TEM samples, and did the imaging and class-summation analysis. EB conceived the study, analyzed data and wrote the manuscript together with PH and AB.

## Conflict of Interest Statement

The authors declare that the research was conducted in the absence of any commercial or financial relationships that could be construed as a potential conflict of interest.
