# Supplementary material for: Cell-Free and Yeast-Based Production of the Malarial Lactate Transporter, PfFNT, Delivers Comparable Yield and Protein Quality
Source: Front Pharmacol. 2019 Apr 10;10:375. doi: 10.3389/fphar.2019.00375 (PMC6467934; doi:10.3389/fphar.2019.00375)
Supplement: Supplementary file 1 [file Data_Sheet_1.PDF]

Cell-free and yeast-based production of the malarial lactate transporter, PfFNT, delivers comparable yield and protein quality

P. Hajek, A. Bader, F. Helmstetter, B. Henke, P. Arnold, E. Beitz

(A) pIVEX2.3 PfFNTopt

tcgcgcgtttcggtgatgacggtgaaaacctctgacacatgcagctcccggagacggtcacagcttgtctgtaagcggatgcc
gggagcagacaagcccgtcagggcgcgtcagcgggtgttggcgggtgtcggggctggcttaactatgcggcatcagagcagat
tgtactgagagtgcaccatcatatgcggtgtgaaataccgcacagatgcgtaaggagaaaataccgcatcaggcgccattcgc
cattcaggctgcgcaactgttgggaagggcgatcggtgcgggcctcttcgctattacgccagctggcgaaagggggatgtgct
gcaaggcgattaagttgggtaacgccagggttttcccagtcacgacgttgtaaaacgacggccagtgccaagcttgcatgcaa
ggagatggcgcccaacagtcccccggccacggggcctgccaccatacccacgccgaaacaagcgctcatgagcccgaagtggc
gagcccgatcttccccatcggtgatgtcggcgatataggcgccagcaaccgcacctgtggcgccggtgatgccggccacgatg
cgtccggcgtagaggatcgagatctcgatcccgcgaaattaatacgactcactatagggagaccacaacggtttccctctaga
aataattttgtttaactttaagaaggagatatacatatgATGTACCCATACGACGTTCCTGACTACGCGGACACTAGTATGCC
TCCCAACAACTCGAAATATGTTCTTGACCCTGTTTCTATCAAAAGCGTGTGCGGAGGGGAAGAAAGCTATATCCGTTGTGTTG
AGTATGGCAAGAAGAAAGCCCATTACAGTAATCTGAATCTTTTAGCCAAAGCTATTCTGGCAGGGATGTTCGTAGGCCTGTGT
GCACATGCGTCAGGAATCGCGGGTGGGTTGTTCTACTACCACAAACTGCGTGAGATTGTTGGAGCGTCTATGAGTGTCTTTGT
GTACGGCTTTACATTTCCGATAGCATTCATGTGCATCATCTGTACCGGTTCGGATTTGTTTACCGGTAATACACTAGCGGTCA
CAATGGCACTGTACGAAAAGAAAGTTAAACTTTTAGACTATCTCCGGGTTATGACCATCTCGTTATTTGGGAACTATGTCGGT
GCAGTCTCATTTGCTTTCTTTGTGTCCTACTTGTCGGGTGCTTTCACTAACGTGCATGCTGTGGAAAAGAATCATTTCTTTCA
GTTTCTGAATGATATCGCAGAGAAGAAAGTTCACCATACATTTGTGGAATGCGTTAGCTTAGCCGTGGGTTGTAACATATTTG
TATGCTTGGCGGTGTATTTCGTGCTGACTCTCAAAGATGGGGCTGGGTATGTATTTTCTGTGTTCTTTGCAGTTTATGCCTTT
GCGATTGCCGGCTATGAGCATATCATAGCCAACATCTATACCCTGAATATAGCCCTGATGGTGAATACGAAGATTACGGTGTA
TCAGGCCTATATTAAAAACCTGCTGCCGACACTGTTGGGCAACTACATTGCGGGTGCGATTGTCCTGGGACTGCCGCTGTATT
TCATTTACAAAGAACACTACTACAACTTCGAACGCAGTAAACGCGACAACAATGATGCGCAAATGAAATCCCTGAGCATTGAA
CTGCGCAACctcgagCACCACCACCACCACCACCATCATCATCATtgagatccggctgctaacaaagcccgaaaggaagctga
gttggctgctgccaccgctgagcaataactagcataaccccttggggcctctaaacgggtcttgaggggttttttgctgaaag
gaggaactatatccggatatccacaggacgggtgtggtcgccatgatcgcgtagtcgatagtggctccaagtagcgaagcgag
caggactgggcggcggccaaagcggtcggacagtgctccgagaacgggtgcgcatagaaattgcatcaacgcatatagcgcta
gcagcacgccatagtgactggcgatgctgtcggaatggacgatatcccgcaagaggcccggcagtaccggcataaccaagcct
atgcctacagcatccagggtgacggtgccgaggatgacgatgagcgcattgttagatttcatacacggtgcctgactgcgtta
gcaatttaactgtgataaactaccgcattaaagcttatcgatgataagctgtcaaacatgagaattcgtaatcatgtcatagc
tgtttcctgtgtgaaattgttatccgctcacaattccacacaacatacgagccggaagcataaagtgtaaagcctggggtgcc
taatgagtgagctaactcacattaattgcgttgcgctcactgcccgctttccagtcgggaaacctgtcgtgccagctgcatta
atgaatcggccaacgcgcggggagaggcggtttgcgtattgggcgctcttccgcttcctcgctcactgactcgctgcgctcgg
tcgttcggctgcggcgagcggtatcagctcactcaaaggcggtaatacggttatccacagaatcaggggataacgcaggaaag
aacatgtgagcaaaaggccagcaaaaggccaggaaccgtaaaaaggccgcgttgctggcgtttttccataggctccgcccccc
tgacgagcatcacaaaaatcgacgctcaagtcagaggtggcgaaacccgacaggactataaagataccaggcgtttccccctg
gaagctccctcgtgcgctctcctgttccgaccctgccgcttaccggatacctgtccgcctttctcccttcgggaagcgtggcg
ctttctcatagctcacgctgtaggtatctcagttcggtgtaggtcgttcgctccaagctgggctgtgtgcacgaaccccccgt
tcagcccgaccgctgcgccttatccggtaactatcgtcttgagtccaacccggtaagacacgacttatcgccactggcagcag
ccactggtaacaggattagcagagcgaggtatgtaggcggtgctacagagttcttgaagtggtggcctaactacggctacact
agaaggacagtatttggtatctgcgctctgctgaagccagttaccttcggaaaaagagttggtagctcttgatccggcaaaca
aaccaccgctggtagcggtggtttttttgtttgcaagcagcagattacgcgcagaaaaaaaggatctcaagaagatcctttga
tcttttctacggggtctgacgctcagtggaacgaaaactcacgttaagggattttggtcatgagattatcaaaaaggatcttc
acctagatccttttaaattaaaaatgaagttttaaatcaatctaaagtatatatgagtaaacttggtctgacagttaccaatg
cttaatcagtgaggcacctatctcagcgatctgtctatttcgttcatccatagttgcctgactccccgtcgtgtagataacta
cgatacgggagggcttaccatctggccccagtgctgcaatgataccgcgagacccacgctcaccggctccagatttatcagca
ataaaccagccagccggaagggccgagcgcagaagtggtcctgcaactttatccgcctccatccagtctattaattgttgccg
ggaagctagagtaagtagttcgccagttaatagtttgcgcaacgttgttgccattgctacaggcatcgtggtgtcacgctcgt
cgtttggtatggcttcattcagctccggttcccaacgatcaaggcgagttacatgatcccccatgttgtgcaaaaaagcggtt
agctccttcggtcctccgatcgttgtcagaagtaagttggccgcagtgttatcactcatggttatggcagcactgcataattc
tcttactgtcatgccatccgtaagatgcttttctgtgactggtgagtactcaaccaagtcattctgagaatagtgtatgcggc
gaccgagttgctcttgcccggcgtcaatacgggataataccgcgccacatagcagaactttaaaagtgctcatcattggaaaa
cgttcttcggggcgaaaactctcaaggatcttaccgctgttgagatccagttcgatgtaacccactcgtgcacccaactgatc
ttcagcatcttttactttcaccagcgtttctgggtgagcaaaaacaggaaggcaaaatgccgcaaaaaagggaataagggcga
cacggaaatgttgaatactcatactcttcctttttcaatattattgaagcatttatcagggttattgtctcatgagcggatac
atatttgaatgtatttagaaaaataaacaaataggggttccgcgcacatttccccgaaaagtgccacctgacgtctaagaaac
cattattatcatgacattaacctataaaaataggcgtatcacgaggccctttcgtc

Cell-free and yeast-based production of the malarial lactate transporter, PfFNT, delivers comparable yield and protein quality

P. Hajek, A. Bader, F. Helmstetter, B. Henke, P. Arnold, E. Beitz

(B) pPink-HC PfFNTopt

```
agatctaacatccaaagacgaaaggttgaatgaaacctttttgccatccgacatccacaggtccattctcacacataagtgcc
aaacgcaacaggaggggatacactagcagcagaccgttgcaaacgcaggacctccactcctcttctcctcaacacccactttt
gccatcgaaaaaccagcccagttattgggcttgattggagctcgctcattccaattccttctattaggctactaacaccatga
ctttattagcctgtctatcctggcccccctggcgaggttcatgtttgtttatttccgaatgcaacaagctccgcattacaccc
gaacatcactccagatgagggctttctgagtgtggggtcaaatagtttcatgttccccaaatggcccaaaactgacagtttaa
acgctgtcttggaacctaatatgacaaaagcgtgatctcatccaagatgaactaagtttggttcgttgaaatgctaacggcca
gttggtcaaaaagaaacttccaaaagtcggcataccgtttgtcttgtttggtattgattgacgaatgctcaaaaataatctca
ttaatgcttagcgcagtctctctatcgcttctgaaccccggtgcacctgtgccgaaacgcaaatggggaaacacccgctttttt
ggatgattatgcattgtctccacattgtatgcttccaagattctggtgggaatactgctgatagcctaacgttcatgatcaaa
atttaactgttctaacccctacttgacagcaatatataaacagaaggaagctgccctgtcttaaaccttttttttttatcatca
ttattagcttactttcataattgcgactggttccaattgacaagcttttgattttaacgacttttaacgacaacttgagaaga
tcaaaaaacaactaattattcgaaacggaattcATGCCTCCCAACAACTCGAAATATGTTCTTGACCCTGTTTCTATCAAAAG
CGTGTGCGGAGGGGAAGAAAGCTATATCCGTTGTGTTGAGTATGGCAAGAAGAAAGCCCATTACAGTAATCTGAATCTTTTAG
CCAAAGCTATTCTGGCAGGGATGTTCGTAGGCCTGTGTGCACATGCGTCAGGAATCGCGGGTGGGTTGTTCTACTACCACAAA
CTGCGTGAGATTGTTGGAGCGTCTATGAGTGTCTTTGTGTACGGCTTTACATTTCCGATAGCATTCATGTGCATCATCTGTAC
CGGTTCGGATTTGTTTACCGGTAATACACTAGCGGTCACAATGGCACTGTACGAAAAGAAAGTTAAACTTTTAGACTATCTCC
GGGTTATGACCATCTCGTTATTTGGGAACTATGTCGGTGCAGTCTCATTTGCTTTCTTTGTGTCCTACTTGTCGGGTGCTTTC
ACTAACGTGCATGCTGTGGAAAAGAATCATTTCTTTCAGTTTCTGAATGATATCGCAGAGAAGAAAGTTCACCATACATTTGT
GGAATGCGTTAGCTTAGCCGTGGGTTGTAACATATTTGTATGCTTGGCGGTGTATTTCGTGCTGACTCTCAAAGATGGGGCTG
GGTATGTATTTTCTGTGTTCTTTGCAGTTTATGCCTTTGCGATTGCCGGCTATGAGCATATCATAGCCAACATCTATACCCTG
AATATAGCCCTGATGGTGAATACGAAGATTACGGTGTATCAGGCCTATATTAAAAACCTGCTGCCGACACTGTTGGGCAACTA
CATTGCGGGTGCGATTGTCCTGGGACTGCCGCTGTATTTCATTTACAAAGAACACTACTACAACTTCGAACGCAGTAAACGCG
ACAACAATGATGCGCAAATGAAATCCCTGAGCATTGAACTGCGCAACctcgagATCGAAGGTCGTCACCACCACCACCACCAC
CATCATCATCATtgataatagggtaccggccggccatttaaatacaggccccttttcctttgtcgatatcatgtaattagtta
tgtcacgcttacattcacgccctcctcccacatccgctctaaccgaaaaggaaggagttagacaacctgaagtctaggtccct
atttatttttttaatagttatgttagtattaagaacgttatttatatttcaaatttttcttttttttctgtacaaacgcgtg
tacgcatgtaacattatactgaaaaccttgcttgagaaggttttgggacgctcgaaggctttaatttgcaagctggatccgcg
gccgccttccaaactctcatggattctcaggtaataggtattctaggaggaggccagctaggccgaatgattgttgaggccgc
tagcaggctcaatatcaagaccgtgattcttgatgatggtttttcacctgctaagcacattaatgctgcgcaagaccacatcg
acggatcattcaaagatgaggaggctatcgccaagttagctgccaaatgtgatgttctcactgtagagattgagcatgtcaac
acagatgctctaaagagagttcaagacagaactggaatcaagatatatcctttaccagagacaatcgaactaatcaaggataa
gtacttgcaaaaggaacatttgatcaagcacaacatttcggtgacaaagtctcagggtatagaatctaatgaaaaggcgctgc
ttttgtttggagaagagaatggatttccatatctgttgaagtcccggactatggcttatgatggaagaggcaattttgtagtg
gagtctaaagaggacatcagtaaggcattagagttcttgaaagatcgtccattgtatgccgagaagtttgctccttttgttaa
agaattagcggtaatggttgtgagatcactggaaggcgaagtattctcctacccaaccgtagaaactgtgcacaaggacaata
tctgtcatattgtgtatgctccggccagagttaatgacaccatccaaaagaaagctcaaatattagctgaaaacactgtgaag
actttcccaggcgctggaatcttcggagttgagatgttcctattgtctgatggagaacttcttgtaaatgagattgctccaag
gccccacaattctggtcactatacaatcgatgcatgtgtaacatctcagttcgaagcacatgtaagagccataactggtctgc
caatgccactagatttcaccaaactatctacttccaacaccaacgctattatgctcaatgttttgggtgctgaaaaatctcac
ggggaattagagttttgtagaagagccttagaaacacccggtgcttctgtatatctgtacggaaagaccacccgattggctcg
taagatgggtcatatcaacataataggatcttccatgttggaagcagaacaaaagttagagtacattctagaagaatcaaccc
acttaccatccagtactgtatcagctgacactaaaccgttggttggagttatcatgggttcagactctgatctacctgtgatt
tcgaaaggttgcgatattttaaaacagtttggtgttccattcgaagttactattgtctctgctcatagaacaccacagagaat
gaccagatatgcctttgaagccgctagtagaggtatcaaggctatcattgcaggtgctggtggtgctgctcatcttccaggaa
tggttgctgccatgactccgttgccagtcattggtgttcctgtcaagggctctacgttggatggtgtagactcgctacactcg
attgtccaaatgcctagaggtgttcctgtggctacggttgctatcaacaacgccaccaatgccgctctgttggccatcaggat
tttaggtacaattgaccacaaatggcaaaaggaaatgtccaagtatatgaatgcaatggagaccgaagtgttggggaaggcat
ccaacttggaatctgaagggtatgaatcctatttgaagaatcgtctttgaatttagtattgtttttaatagatgtatatata
atagtacacgtaacttatctattccattcataattttattttaaaggttcggtagaaatttgtcctccaaaaagttggttaga
gcctggcagttttgataggcattattatagattgggtaatatttaccctgcacctggaggaactttgcaaagagcctcatgtg
cggcgcgccaggccataatggccaaacggtttctcaattactatatactactaaccatttacctgtagcgtatttcttttccc
tcttcgcgaaagctcaagggcatcttcttgactcatgaaaaatatctggatttcttctgacagatcatcacccttgagcccaa
ctctctagcctatgagtgtaagtgatagtcatcttgcaacagattattttggaacgcaactaacaaagcagatacacccttca
gcagaatcctttctggatattgtgaagaatgatcgccaaagtcacagtcctgagacagttcctaatctttaccccatttacaa
gttcatccaatcagacttcttaacgcctcatctggcttatatcaagcttaccaacagttcagaaactcccagtccaagtttct
tgcttgaaagtgcgaagaatggtgacaccgttgacaggtacacctttatgggacattcccccagaaaaataatcaagactggg
cctttagagggtgctgaagttgaccccttggtgcttctggaaaaagaactgaagggcaccagacaagcgcaacttcctggtat
tcctcgtctaagtggtggtgccataggatacatctcgtacgattgtattaagtactttgaaccaaaaactgaaagaaaactga
aagatgttttgcaacttccggaagcagctttgatgttgttcgacacgatcgtggcttttgacaatgtttatcaaagattccag
gtaattggaaacgtttctctatccgttgatgactcggacgaagctattcttgagaaatattataagacaagagaagaagtgga
aaagatcagtaaagtggtatttgacaataaaactgttccctactatgaacagaaagatattattcaaggccaaacgttcacct
ctaatattggtcaggaagggtatgaaaaccatgttcgcaagctgaaagaacatattctgaaaggagacatcttccaagctgtt
ccctctcaaagggtagccaggccgacctcattgcaccctttcaacatctatcgtcatttgagaactgtcaatccttctccata
```

Cell-free and yeast-based production of the malarial lactate transporter, PfFNT, delivers comparable yield and protein quality

P. Hajek, A. Bader, F. Helmstetter, B. Henke, P. Arnold, E. Beitz

```
catgttctatattgactatctagacttccaagttgttggtgcttcacctgaattactagttaaatccgacaacaacaacaaaa
tcatcacacatcctattgctggaactcttcccagaggtaaaactatcgaagaggacgacaattatgctaagcaattgaagtcg
tctttgaaagacagggccgagcacgtcatgctggtagatttggccagaaatgatattaaccgtgtgtgtgagcccaccagtac
cacggttgatcgtttattgactgtggagagattttctcatgtgatgcatcttgtgtcagaagtcagtggaacattgagaccaa
acaagactcgcttcgatgctttcagatccattttcccagcaggaaccgtctccggtgctccgaaggtaagagcaatgcaactc
ataggagaattggaaggagaaaagagaggtgtttatgcgggggccgtaggacactggtcgtacgatggaaaatcgatggacac
atgtattgccttaagaacaatggtcgtcaaggacggtgtcgcttaccttcaagccggaggtggaattgtctacgattctgacc
cctatgacgagtacatcgaaaccatgaacaaaatgagatccaacaataacaccatcttggaggctgagaaaatctggaccgat
aggttggccagagacgagaatcaaagtgaatccgaagaaaacgatcaatgaacggaggacgtaagtaggaatttatggtttgg
ccataatggcctagcttggcgtaatcatggtcatagctgtttcctgtgtgaaattgttatccgctcacaattccacacaacat
acgagccggaagcataaagtgtaaagcctggggtgcctaatgagtgagctaactcacattaattgcgttgcgctcactgcccg
ctttccagtcgggaaacctgtcgtgccagctgcattaatgaatcggccaacgcgcggggagaggcggtttgcgtattgggcgc
tcttccgcttcctcgctcactgactcgctgcgctcggtcgttcggctgcggcgagcggtatcagctcactcaaaggcggtaat
acggttatccacagaatcaggggataacgcaggaaagaacatgtgagcaaaaggccagcaaaaggccaggaaccgtaaaaagg
ccgcgttgctggcgtttttccataggctccgcccccctgacgagcatcacaaaaatcgacgctcaagtcagaggtggcgaaac
ccgacaggactataaagataccaggcgtttccccctggaagctccctcgtgcgctctcctgttccgaccctgccgcttaccgg
atacctgtccgcctttctcccttcgggaagcgtggcgctttctcatagctcacgctgtaggtatctcagttcggtgtaggtcg
ttcgctccaagctgggctgtgtgcacgaaccccccgttcagcccgaccgctgcgccttatccggtaactatcgtcttgagtcc
aacccggtaagacacgacttatcgccactggcagcagccactggtaacaggattagcagagcgaggtatgtaggcggtgctac
agagttcttgaagtggtggcctaactacggctacactagaaggacagtatttggtatctgcgctctgctgaagccagttacct
tcggaaaaagagttggtagctcttgatccggcaaacaaaccaccgctggtagcggtggtttttttgtttgcaagcagcagatt
acgcgcagaaaaaaaggatctcaagaagatcctttgatcttttctacggggtctgacgctcagtggaacgaaaactcacgtta
agggattttggtcatgagattatcaaaaaggatcttcacctagatccttttaaattaaaaatgaagttttaaatcaatctaaa
gtatatatgagtaaacttggtctgacagttaccaatgcttaatcagtgaggcacctatctcagcgatctgtctatttcgttca
tccatagttgcctgactccccgtcgtgtagataactacgatacgggagggcttaccatctggccccagtgctgcaatgatacc
gcgagacccacgctcaccggctccagatttatcagcaataaaccagccagccggaagggccgagcgcagaagtggtcctgcaa
ctttatccgcctccatccagtctattaattgttgccgggaagctagagtaagtagttcgccagttaatagtttgcgcaacgtt
gttgccattgctacaggcatcgtggtgtcacgctcgtcgtttggtatggcttcattcagctccggttcccaacgatcaaggcg
agttacatgatcccccatgttgtgcaaaaaagcggttagctccttcggtcctccgatcgttgtcagaagtaagttggccgcag
tgttatcactcatggttatggcagcactgcataattctcttactgtcatgccatccgtaagatgcttttctgtgactggtgag
tactcaaccaagtcattctgagaatagtgtatgcggcgaccgagttgctcttgcccggcgtcaatacgggataataccgcgcc
acatagcagaactttaaaagtgctcatcattggaaaacgttcttcggggcgaaaactctcaaggatcttaccgctgttgagat
ccagttcgatgtaacccactcgtgcacccaactgatcttcagcatcttttactttcaccagcgtttctgggtgagcaaaaaca
ggaaggcaaaatgccgcaaaaaagggaataagggcgacacggaaatgttgaatactcatactcttcctttttcaatattattg
aagcatttatcagggttattgtctcatgagcggatacatatttgaatgtatttagaaaaataaacaaataggggttccgcgca
catttccccgaaaagtgccacctgacgtctaagaaaccattattatcatgacattaacctataaaaataggcgtatcacgagg
ccctttcgtctcgcgcgtttcggtgatgacggtgaaaacctctgacacatgcagctcccggagacggtcacagcttgtctgta
agcggatgccgggagcagacaagcccgtcagggcgcgtcagcgggtgttggcgggtgtcggggctggcttaactatgcggcat
cagagcagattgtactgagagtgcaccatatgcggtgtgaaataccgcacagatgcgtaaggagaaaataccgcatcaggcgc
cattcgccattcaggctgcgcaactgttgggaagggcgatcggtgcgggcctcttcgctattacgccagctggcgaaaggggg
atgtgctgcaaggcgattaagttgggtaacgccagggttttcccagtcacgacgttgtaaaacgacggccagtgaattg
```

Supplementary Fig. S1. DNA sequences of the used expression constructs. (A) Cell-free PfFNT was transcribed from the pIVEX2.3 plasmid. (B) *P. pastoris* PfFNT was transcribed from the pPink-HC plasmid. The plasmid sequences are shown in lower case, the coding regions in upper case.

Cell-free and yeast-based production of the malarial lactate transporter, PfFNT, delivers comparable yield and protein quality

P. Hajek, A. Bader, F. Helmstetter, B. Henke, P. Arnold, E. Beitz

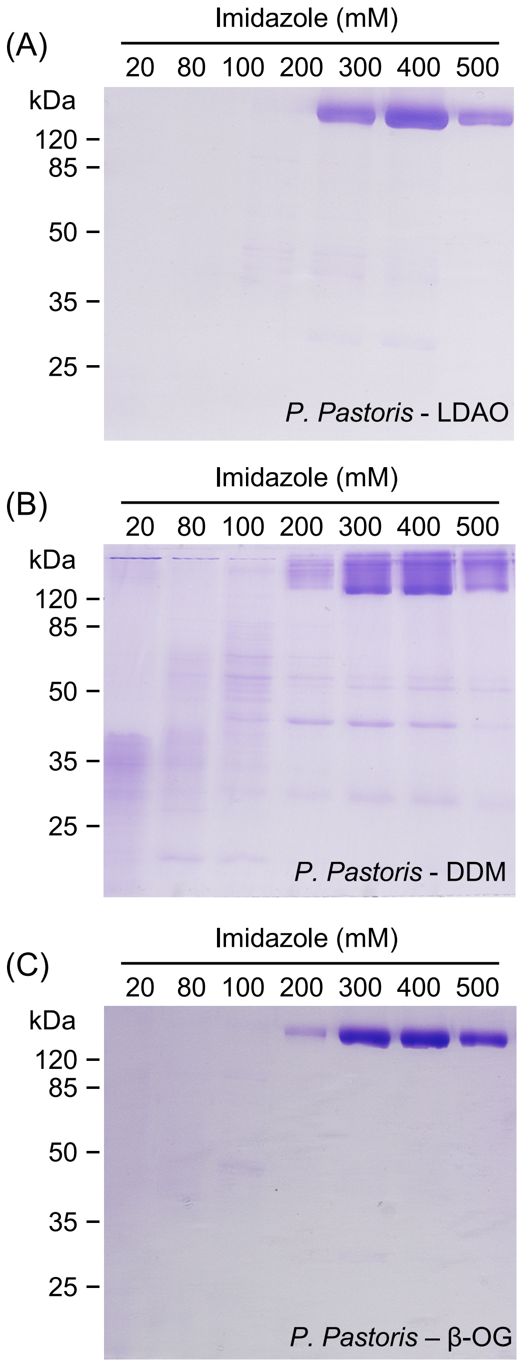

Supplementary Fig. S2. Ni^{2+}-affinity purification of *P. pastoris* expressed PfFNT in LDAO (A), DDM (B), and β-OG (C) via C-terminal His-tags. PfFNT monomers have a molecular mass of 30 kDa, the pentamer accordingly of 150 kDa.

Cell-free and yeast-based production of the malarial lactate transporter, PfFNT, delivers comparable yield and protein quality

P. Hajek, A. Bader, F. Helmstetter, B. Henke, P. Arnold, E. Beitz

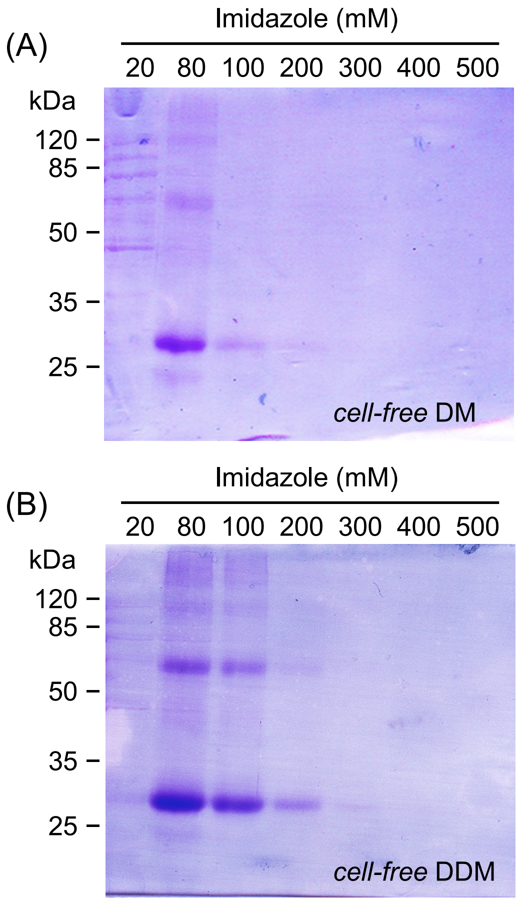

Supplementary Fig. S3. Detergent exchange during Ni^{2+}-affinity chromatography from Brij78 to DM (A) and DDM (B) of cell-free produced PfFNT. Monomers have a molecular mass of 30 kDa, the pentamer accordingly of 150 kDa. The laddering indicates partially SDS-resistant homooligomers up to the pentamer.

Cell-free and yeast-based production of the malarial lactate transporter, PfFNT, delivers comparable yield and protein quality

P. Hajek, A. Bader, F. Helmstetter, B. Henke, P. Arnold, E. Beitz

(A) (B)

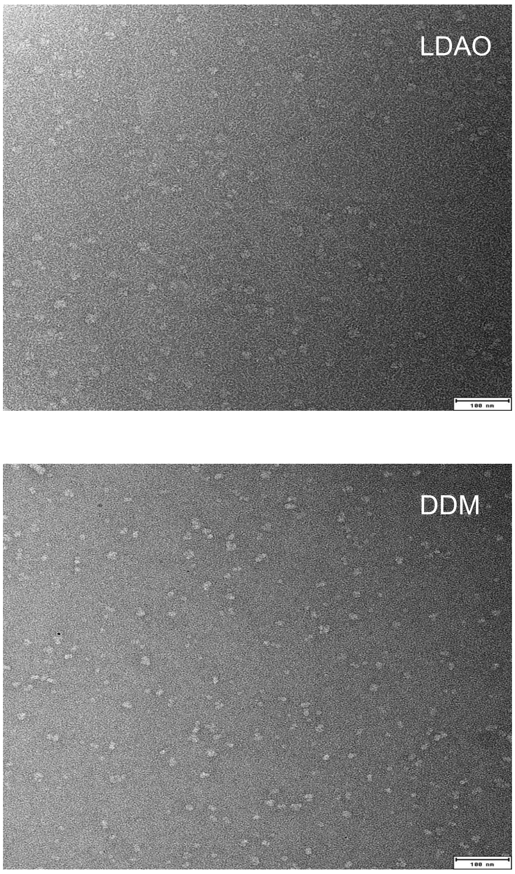

Supplementary Fig. S4. Transmission electron microscopy of *P. pastoris* expressed PfFNT in LDAO (A) and DDM (B). Both detergents led to blurry particle images and were inferior to DM (see Fig. 2 of the main paper).
